# Investigation of Nonlinear Pupil Dynamics by Recurrence Quantification Analysis

**DOI:** 10.1155/2013/420509

**Published:** 2013-09-26

**Authors:** L. Mesin, A. Monaco, R. Cattaneo

**Affiliations:** ^1^Mathematical Biology and Physiology Group, Department of Electronics and Telecommunications, Politecnico di Torino, Torino 10129, Italy; ^2^Department of Health Sciences, Università di L' Aquila, L' Aquila 67010, Italy

## Abstract

Pupil is controlled by the autonomous nervous system (ANS). It shows complex movements and changes of size even in conditions of constant stimulation. The possibility of extracting information on ANS by processing data recorded during a short experiment using a low cost system for pupil investigation is studied. Moreover, the significance of nonlinear information contained in the pupillogram is investigated. We examined 13 healthy subjects in different stationary conditions, considering habitual dental occlusion (HDO) as a weak stimulation of the ANS with respect to the maintenance of the rest position (RP) of the jaw. Images of pupil captured by infrared cameras were processed to estimate position and size on each frame. From such time series, we extracted linear indexes (e.g., average size, average displacement, and spectral parameters) and nonlinear information using recurrence quantification analysis (RQA). Data were classified using multilayer perceptrons and support vector machines trained using different sets of input indexes: the best performance in classification was obtained including nonlinear indexes in the input features. These results indicate that RQA nonlinear indexes provide additional information on pupil dynamics with respect to linear descriptors, allowing the discrimination of even a slight stimulation of the ANS. Their use in the investigation of pathology is suggested.

## 1. Introduction

The dynamics of the pupil shows apparently random movements and changes of size, even in constant conditions of light and visual stimulus. This complex behaviour reflects the action of the autonomous nervous system (ANS) [1]. The study of stress related pathologies is promoting the development of low cost devices for monitoring the physiological systems controlled by the ANS [2, 3] and the study of methods to process images captured from pupil [4].

The contraction and dilation of the pupil are controlled by the two branches of the ANS, specifically by the sympathetic nerve centre (Budge's ciliospinalis centre) and the parasympathetic centre (Edinger-Westphal Nucleus). They promote pupil dilation (mydriasis) and constriction (miosis), respectively.

The oscillation of pupil size varies according to subjective characteristics or to the properties of light stimulation, but the spontaneous oscillation frequency was found to be independent of age, sex, intensity of light, and time of day [5]. 

Under conditions of constant light and vision, some frequencies of oscillation are coupled among various systems affected by the control of the ANS (e.g., cardiovascular and respiratory systems) [6, 7]. The pupil is part of this group of systems, sharing some of the common rhythms [8–12].

The interest, therefore, for the study of spontaneous erratic dynamics of pupil comes from the possibility to monitor in a simple, direct, and easily accessible way the physiological state of the ANS. In this regard, studies have been conducted to investigate the physiology of this system and its involvement in the course of diseases of the ANS [13–17].

Most of the studies considered the dynamics of pupil as linear and steady, so that classical Fourier analysis could be applied [7]. Nevertheless, the presence of nonstationary dynamics in the behaviour of pupil oscillations has been reported in a few studies [18, 19]. The complex dynamics of the signal suggested the necessity to study this system using nonlinear analysis techniques [20]. However, the analysis of spontaneous pupil oscillations by nonlinear techniques has not been deepened in the literature yet. 

Recurrence Quantification Analysis (RQA) is a specific nonlinear technique which was introduced to study the nonlinear dynamics of various natural and artificial systems [21], including biological signals [21–27]. One of the advantages of this technique is the ability to analyze relatively short time series of nonlinear data [28]. 

The purpose of this study is to assess the applicability of RQA to process spontaneous oscillations of the pupil, recorded by a low cost system during short experiments (adequate for a clinical monitoring) in specific conditions of the ANS. Two different stimulations of the ANS are considered: the first is constant light, which is considered as a strong stimulation, deeply affecting pupil dynamics; the second is habitual dental occlusion (HDO) (see Section 2.3), which is expected to elicit a weak response of the ANS [29]. The ability of different linear and nonlinear indexes in discriminating different conditions (in particular, the sensitivity to the weak stimulation by HDO) is tested using statistical analysis and multi-index classification.

## 2. Materials and Methods 

### 2.1. Instrumentation

Images of the pupils were acquired by the Oculus system (Inventis SRL, Padova, Italy), using two infrared CCD cameras (resolution 720 × 576 pixels, 256 grey levels) mounted on a light helmet (1.5 kg), with sampling frequency of 25 frame/s. The eyes were illuminated with an infrared diode with 880 nm of wave length; moreover, during experiments on pupil dynamics under constant light conditions, illumination was provided by green LEDs (one for each eye) with 540 nm of wave length and intensity of 1.5 mcd.

### 2.2. Experimental Setup

The subjects were lying supine on a bed for clinical examination. The environment was kept at a constant temperature of 21°C and with relative humidity of 50%. Causes of alarm (fixed and mobile phones, speakers, bells, etc.) were excluded. The recording session was preceded by 3 minutes of environmental adaptation of the subject. Then, the helmet was applied and was maintained until the end of the recording without further handling. 

The correct procedure and execution of tests was first explained to the subject. Then, some brief tests were proposed, in order to be sure that the instructions were well understood. This phase took about 2 minutes.

Two operators assisted the subjects during the experiments. The first took care of the subject (pretest and test instructions, helmet handling, check on the correctness of execution), and the second controlled data acquisition.

### 2.3. Experiments

Biocular, one minute long acquisitions were obtained from 13 young, healthy subjects (aged 27.1 ± 6.9; 5 females, 8 males). The tonic adapted size of the two pupils was investigated in darkness and constant light conditions. In light condition, the subjects directed the gaze toward the green LEDs till they obtained the fusion of the two different LEDs. In darkness, subjects were asked to keep the eyes straight ahead. Different stationary conditions were considered, which require a different involvement of the sympathetic and parasympathetic control: neutral position of the jaw (rest position: RP) and HDO. HDO is the full contact of the two dental arches. It is achieved spontaneously during swallowing when the teeth of the jaw and the teeth of the maxilla fit through the respective contact surface. This dental occlusion is also habitual because it is obtained spontaneously and routinely each time an individual decides to close the teeth in complete and full contact with each other. During dental occlusion, the effect of muscle fatigue (which would elicit the autonomic system) was excluded by avoiding prolonged teeth clenching. Attention was paid to check the activity of mimic muscles (reflecting a possible erroneous occlusion). 

RP and HDO were investigated both in light and in darkness, so that four experiments were performed for each subject. The tests were carried out according to a randomized sequence, in order to avoid cumulative effects. One minute of rest was inserted between consecutive tests. During such an interval, the subject's eyes were closed. Subsequently, the subject opened the eyes and, after 30 seconds of adaptation, the following specific test started.

### 2.4. Time Series Extraction

The pupil of each eye was tracked identifying it with the region growing algorithm, which guarantees that a connected region is identified starting from the darkest point in the image. Such a point was selected close to the centre of the pupil identified from the previous image, in order to be sure to exclude other dark portions of the frames (e.g., an eyelash). The border of the identified pupil region was then computed and, finally, it was interpolated with a circle using the analytical method proposed in [30]. 

Pupil size was computed as the sum of pixels identified by the region growing algorithm. Pupil position was given by the centre of the interpolating circle.

Possible mistakes of the processing algorithm were determined by noisy frames or blinks (even if the subjects were asked to keep the eyes open during the 60 s acquisitions). Such mistakes (less than 0.5% of frames for all considered videos) determined rapid, not physiological variations of the estimated dimension and motion of the pupil, so that they could be automatically identified and removed (by cubic interpolation). This filtering is not expected to affect the signals, as the bandwidth of pupil size and movement (if microsaccades are neglected) is lower than a few Hz. 

Images from both eyes were processed to extract the size and the position of the two pupils. The mean size and the mean position (averaging across the two pupils) were considered for further processing.

### 2.5. Time Series Embedding

The estimated pupil area was considered as a time series extracted from a deterministic physiological system. Suppose that such a system can be described by a set of complicated, unknown deterministic rules as

(1) $$\begin{array}{c} \frac{d\vec{x}}{dt}=\vec{F}\left(\vec{x},t\right), \end{array}$$

where $\vec{x}$ is the vector of state variables of the system and $\vec{F}$ is a set of functions called the vector field, defining the evolution of the state variables in time. If the system works in stationary conditions, we can expect that the vector field is not an explicit function of time (i.e., the same deterministic rules are used to control the size of the pupil over time). In such a case, the system is said to be autonomous:

(2) $$\begin{array}{c} \frac{d\vec{x}}{dt}=\vec{F}\left(\vec{x}\right). \end{array}$$

The estimated pupil size can be considered as a time series *y*(*t*) extracted from the system through a measure, which can be modelled by an unknown function *g*(·) of the state variables

(3) $$\begin{array}{c} y\left(t\right)=g\left(\vec{x}\left(t\right)\right). \end{array}$$

The methods of time series embedding [31] were used. Given the single measured time series, multiple information is obtained by considering time delayed versions of the data

(4) $$\begin{array}{c} \vec{Y}\left(t\right)=\left[\begin{array}{c} y\left(t\right)\\ y\left(t-\tau \right)\\ \vdots \\ y\left(t-\left(m-1\right)\tau \right) \end{array}\right], \end{array}$$

where *τ* is a time delay chosen in order that different functions in the vector $\vec{Y}(t)$ contain different information and the number *m* of elements of the vector is called the embedding dimension. The vector $\vec{Y}(t)$ can be considered as a trajectory (parameterized by the time variable *t*) embedded in a space of dimension *m* (phase space). The time delay *τ* was estimated considering the mutual information between the recorded time series and the delayed one as

(5) $$\begin{array}{c} \text{MI}\left(\tau \right)=\int_{U}^{}\int_{V}^{}p_{UV}\left(u,v\right)\;\ln\left(\frac{p_{UV}\left(u,v\right)}{p_{U}\left(u\right)p_{V}\left(v\right)}\right)du\;dv, \end{array}$$

where the time series *y*(*t*) and *y*(*t* − *τ*) are considered as random variables *U* and *V*, respectively, with joint probability density *p*_*UV*_(*u*, *v*) and marginal probabilities *p*_*U*_(*u*) and *p*_*V*_(*v*), respectively. For subsequent analysis, we considered the time delay corresponding to a 90% decrease of mutual information between the maximal value at *τ* = 0 and a reference minimal value (i.e., the average value for large time delays, 100 < *τ* < 200 measured in samples). 

In order to choose the proper embedding dimension, Cao's method [32] was used. It is based on the number of points of the trajectory described by the vector in (4), which are neighbours of other points of the trajectory itself. When the trajectory returns in the vicinity of a point which was passed through before, we say that the system undergoes a recurrence. Notice that the correct estimation of a recurrence is of paramount importance for RQA. When increasing the embedding dimension (adding one element to the vector describing the trajectory), neighbouring points which are close only due to the projection of the trajectory in a low dimensional space (false near neighbours) may turn away. Thus, the number of neighbouring points decreases by increasing the embedding dimension, till false neighbours are present. Real neighbours remain close to each other when increasing further the embedding dimension. Thus, investigating the curve indicating the number of neighbours for different embedding dimensions, it is possible to determine the dimension of the space in which the trajectory in (4) is embedded. Specifically, Cao's method [32] considers the following function of the embedding dimension:

(6) $$\begin{array}{c} E1\left(m\right)=\frac{E\left(m+1\right)}{E\left(m\right)},\\ \text{where}\;\;E\left(m\right)=\frac{1}{N-m\tau }\sum_{i=1}^{N-m\tau }\frac{||y_{i}\left(m+1\right)-y_{n(i,m)}\left(m+1\right)||}{||y_{i}\left(m\right)-y_{n(i,m)}\left(m\right)||}, \end{array}$$

where ||·|| is the absolute distance norm, *y*_*i*_(*m*) is the *i*th reconstructed vector with embedding dimension *m*, and *y*_*n*(*i*,*m*)_(*m*) is the nearest neighbour of *y*_*i*_(*m*) in the *m*-dimensional reconstructed phase space. The function *E*1(*m*) saturates when the correct dimension of the phase space is considered. Thus, such a function has a knee (i.e., a point of maximum curvature), separating a region of increase from a plateau. To estimate automatically the position of the knee, the following procedure was applied. Given a number *N* of tested embedding dimensions, the first (*N*–*M*) values of *E*1(*m*) (with 1 < *M* < *N*) were interpolated by a line, the remaining *M* values by another line. In this way, for each value of *M*, the function was approximated by two lines. The value of *M* providing the minimum mean square error was considered as corresponding to the knee.

The embedding dimension provides an indication of the number of (unknown) state variables of the (unknown) set of deterministic rules describing the dynamics of the system (indicating the complexity of the system). All signals were characterized by a time delay close to 10 and an embedding dimension close to 6. These two values were fixed for all signals, in order to keep the same processing parameters.

### 2.6. Recurrence Quantification Analysis

Recurrence quantification analysis (RQA) is a nonlinear technique providing quantitative indexes related to the number and duration of recurrences of the trajectory of a dynamical system in the phase space. The size of the pupil was considered as a trajectory after applying the time series embedding method described in Section 2.5; the movement of the centre of the pupil was considered as a two-dimensional trajectory. 

All variables provided by RQA are based on the recurrence plot, which is a binary recurrence map obtained by assigning value 1 to the entry (*i*, *j*) if the Euclidean distance between the *i*th and the *j*th point along the trajectory is smaller than a threshold (in which case there is a recurrence of the trajectory) and value 0 otherwise. In this paper, the threshold is chosen to be

(7) $$\begin{array}{c} \text{th}={\left(\frac{{\left(6\sigma \right)}^{m}}{10^{4}}\right)}^{1/m}, \end{array}$$

where *σ* is the standard deviation of the signal. This choice was selected after a fine tuning based on a subset of signals. It can be interpreted as follows: 6*σ* is considered as the essential range of the signal (assumed to vary around its mean plus or minus 3 times its standard deviation) and (6*σ*)^*m*^ is an estimate of the volume of the region spanned by the trajectory in the *m*-dimensional phase space. Such a region is divided into 10^4^ small portions and the threshold th can be considered as the diameter of such small regions sampling the hyper-volume spanned by the trajectory. To exclude neighbouring points which are close in time, the minimum time interval (Theiler window, [31]) between different points considered in the recurrence plot was 10.

### 2.7. Linear and Nonlinear Indexes

The mean size of the pupil was considered as a linear index characterizing pupil dynamics. Moreover, the amplitudes of the movements in the *x* or *y* directions were measured by their standard deviations (STD).

Important linear indexes were extracted using Fourier analysis. Mean frequency (MNF) was computed from the Fourier spectrum of pupil size and movement along the *x* or *y* directions. As further spectral indexes, the percentages of energy of the spectrum of pupil size in the ranges 0.04–0.15 Hz (*F*_Low_) and 0.15–0.5 Hz (*F*_High_) were computed [10]. 

Nonlinear indexes were extracted from the recurrence map. Different indexes can be considered [21], but here we focus only on the two ones which are mostly used in the literature. The recurrence rate is the density of recurrence points

(8) $$\begin{array}{c} \text{RR}=\frac{1}{N^{2}}\sum_{i,j=1}^{N}R\left(i,j\right), \end{array}$$

where *N* is the number of entries of the recurrence map *R*. 

The second considered index is the percentage of determinism, defined as the percentage of recurrence points forming diagonal lines in the recurrence plot of minimal length *L*_min⁡_ (equal to 2 in this work) as

(9) $$\begin{array}{c} \text{DET}=\frac{\sum_{l=L_{\min}}^{N}lP\left(l\right)}{\sum_{i,j=1}^{N}R\left(i,j\right)}, \end{array}$$

where *P*(*l*) is the frequency distribution of the lengths of the diagonal lines. The determinism is related to the predictability of the system (as the length of diagonal lines in the recurrence plot indicates how long neighbouring points remain close) and to Lyapunov exponents (time constants of exponential divergence of close trajectories of a chaotic system [31]).

### 2.8. Signal Processing

The two-sided Wilcoxon signed rank test (considering paired data with Bonferroni correction) was applied to investigate differences between couples of conditions of interest: RP (or HDO) in light compared to RP (or HDO) in darkness, RP compared to HDO, in light or in darkness. The significance level was set to *P* < 0.05. 

Paired statistical analysis makes use of a single index to discriminate between different conditions, but it uses the information that data are paired. A second test was based on checking the discrimination capability of sets of indexes, neglecting the information that data are paired. Different multilayer perceptrons (MLP) and support vector machines (SVM) were trained [33] to learn how to classify RP and HDO conditions (in light or darkness), given the indexes extracted from the data (discriminating light and darkness conditions was not considered, as it is trivial). The classification was performed using all possible sets of 3, 4, or 5 input indexes (with less than 3 indexes, the errors were always large, about 50%; using more than 5 indexes would not be correct, due to the small set of data).

A set of MLP was considered with a single hidden layer (with neurons with hyperbolic tangent activation function) and a single output neuron (with logistic activation function). The number of hidden neurons was varied between 4 and 20. Each network was trained on a subset of 20 data (training set) using the quasi-Newton algorithm for a number of iterations between 2 and 500. The MLP with the best generalization to a subset of 5 data (validation set) was chosen to estimate a single test sample (approximating the output to the closest integer to determine the estimated class). The classification of the same test sample was estimated 9 times, considering the optimal MLP trained and validated on different, randomly chosen, training and validation sets. The class that was chosen the majority number of times was finally considered as the classification of the test sample.

Similarly, different sets of data were used to train an SVM to perform a binary classification, discriminating between RP and HDO conditions. As the classes were not linearly separable, the input space was mapped into a feature space using a polynomial kernel. The order of the kernel was chosen in the range of 2–8 as that guaranteeing the best generalisation on a subset of 5 unseeing data (validation set) after training of a subset of 20 data (with 8 random choices of training and validation set). The selected SVM was then used to classify the single test data left out.

The procedure was repeated for both MLP and SVM classifiers considering as test sample each of the 26 data (RP and HDO conditions, in light or darkness), with a leave-one-out approach. The discrepancy between estimated and actual class was used to quantify the goodness of a specific choice of input features in discriminating data collected in RP or HDO.

The indexes allowing best classification in the leave-one-out test were further used as inputs for classifiers applied to more than a single test sample. The classifiers were again optimized on training and validation sets (using all data excluding those used for test) and then applied to estimate the RP or HDO conditions of unseen 2–6 test data.

## 3. Results

**Figure 1:**
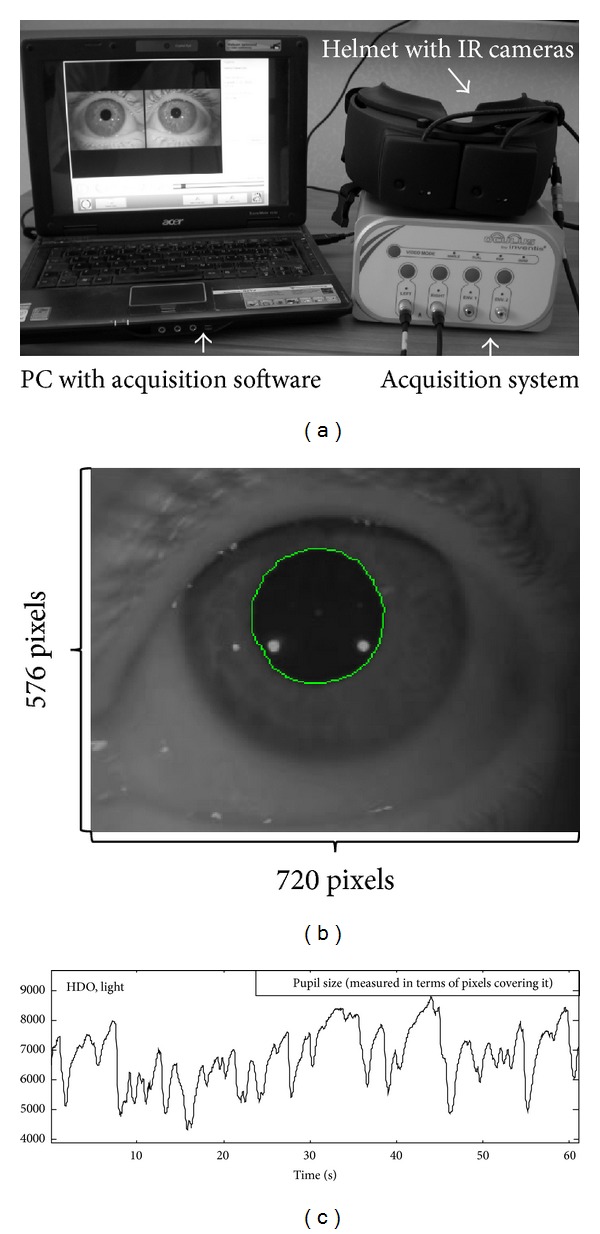
Example of detection and preliminary processing of data. (a) Detection system. (b) Example of processing of a single frame of the video captured by one of the two cameras of the system. The boundary of the pupil is identified and interpolated with a circle. (c) Area of the pupil as a function of time.

Figure 1 shows a representation of the experimental protocol and data preliminary processing. The detection system is shown in Figure 1(a). Two infrared cameras capture images from the eyes when stimulated by a green LED (light conditions) or when they are only illuminated by infrared light (darkness condition). A single frame of the video captured by one of the two cameras of the system is shown in Figure 1(b). The pupil is identified by the region growing algorithm. The boundary is then estimated (and interpolated by a circle, to compute the movements of pupil; see Section 2.4). The pupil area was computed for each frame as the number of pixels covering it and represented as a function of time in Figure 1(c).

**Figure 2:**
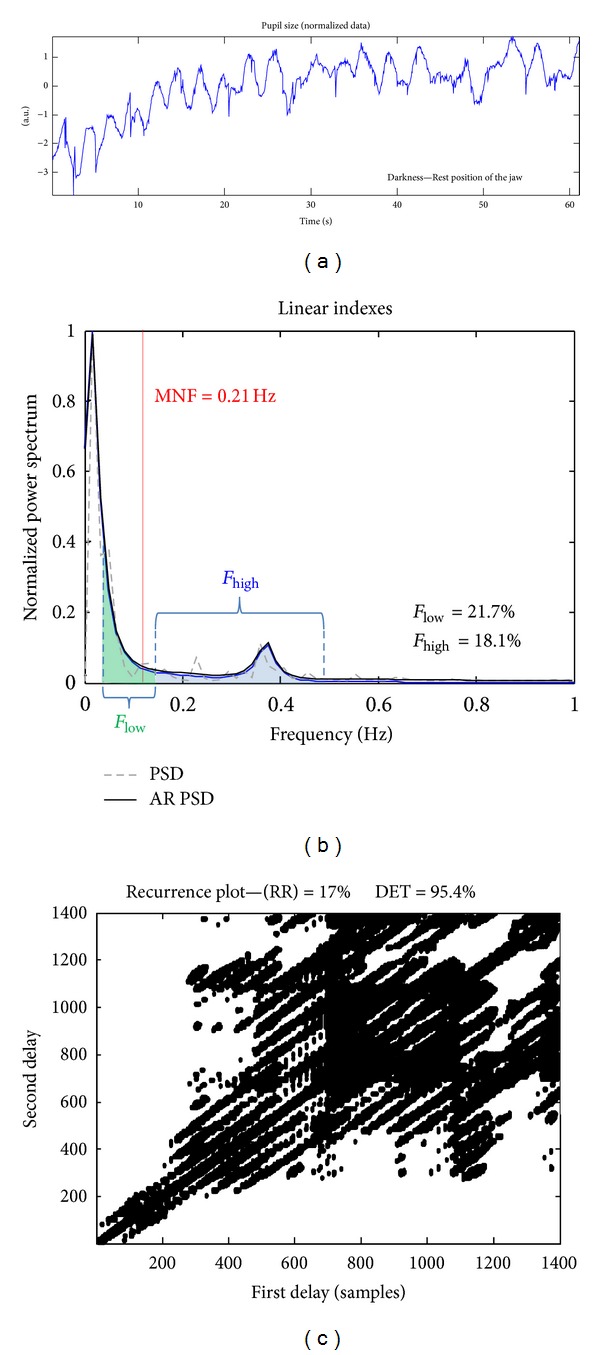
Example of processing of a pupil size time series recorded from a subject in stationary conditions. Normalized data are shown in (a). The extraction of some linear and nonlinear indexes is shown in (b) and (c), respectively (each point in (c) indicates a recurrence).

Figure 2 shows an example of processing of a pupil size time series recorded from a subject in stationary conditions. The estimated time series is shown in Figure 2(a). Data are normalised, removing the mean and dividing by the standard deviation. The extraction of some linear and nonlinear indexes are shown in Figures 2(b) and 2(c), respectively. Linear indexes from Fourier analysis are obtained from the power spectrum (both the spectrum and the estimation by the Yule-Walker autoregressive algorithm [34] are shown; the power spectrum provided by FFT was used in this paper to estimate the considered linear spectral indexes). Nonlinear indexes (RR and DET) are extracted from the recurrence plot, which is shown in Figure 2(c). The number of black points (indicating recurrences) and their distribution along diagonals provide a visual indication of recurrence and determinism of the data. Note that the recurrence plot is symmetric with respect to the diagonal, by definition. The black points are distributed close to the diagonal on the bottom-left portion, indicating an initial transient. On the upper-right region, black points are more diffused, reflecting the pseudoperiodicity of the data.

**Figure 3:**
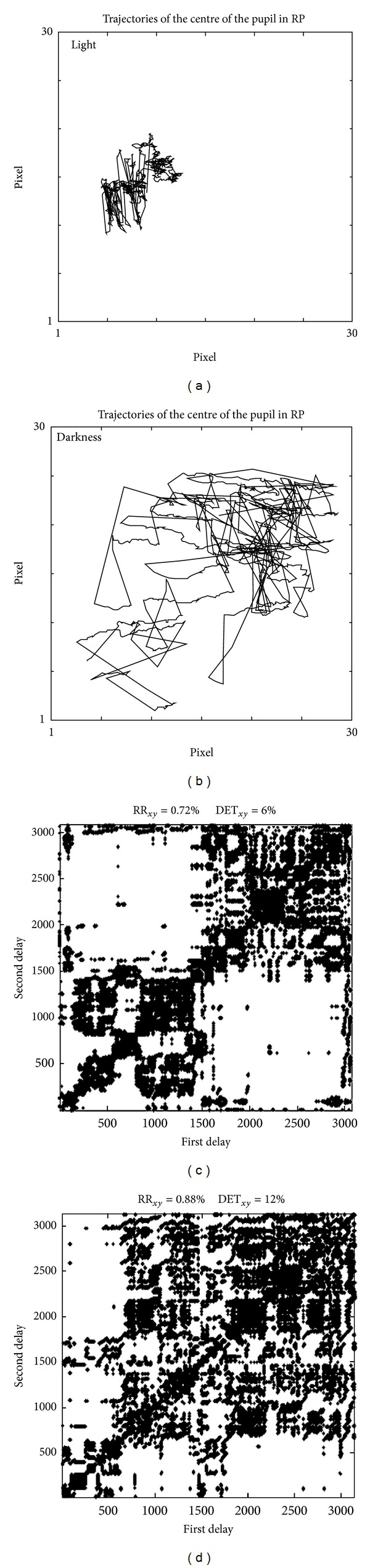
Example of nonlinear processing of pupil movements during (a-c) light and (b-d) darkness condition, recorded from the same subject in RP.

Figure 3 shows an example of processing of pupil movement signals recorded from a single subject in light and darkness stationary conditions. Notice that movements are broader in darkness. The recurrence plot is shown and nonlinear RQA indexes are indicated. Recurrence rate and determinism are very low, indicating that eye movements are erratic. 

Table 1 provides mean and standard deviation of all indexes.  Table 2 indicates the result of the statistical paired test for the significance of differences between interesting conditions. Discriminating between light and darkness condition is surely possible. Discriminating between RP and HDO conditions from pupil dynamics is more difficult, as the stimulation of the ANS during HDO is weak. Statistical analysis of paired data suggests that the two conditions can be discriminated by a few indexes only in dark conditions, whereas the distinction in light is fairly difficult. As mentioned in Section 1, this is in line with our expectations, as light is a much stronger stimulation than changing slightly the position of the jaw, so that it obscures the effect of the latter.

Tables 3 and 4 show the result of the multi-index classification by MLPs and SVMs of RP and HDO, in light and darkness condition, respectively. Again, in light condition, the classification is very difficult (with better performance using SVMs). With MLPs, only a few choices of input features allow obtaining a classification error lower than that of a random classifier (for which 50% error is expected). With SVMs, best classifiers have about 30% of errors. Moreover, the classifier does not benefit from including more input features, indicating that an additional input provides more noise than information. 

When signals are detected in dark conditions, classification is possible. Performances increase slightly by providing more input features with MLPs, whereas they are constant with SVMs. The extent of the vertical movement of the eye (measured by STD_*y*_) and the mean size are the features which are most included in the best sets of input indexes, both considering MLPs and SVMs. Nonlinear indexes extracted by RQA from pupil size time series are always included. In particular, both RR and DET are selected in the optimal sets of 4 and 5 input sets using MLPs, together with the above mentioned features (size and STD_*y*_), indicating the importance of including the considered nonlinear information to improve classification (reflecting a better description and characterization of pupil dynamics).

 In order to better underline the contribution of nonlinear information, the maximum performances obtained using only linear indexes were as follows: 42% and 38% of error using 3 inputs with MLP and SVM, respectively; 42% and 34% of error using 4 inputs with MLP and SVM, respectively; 46% and 42% of error using 5 inputs with MLP and SVM, respectively. The errors are very high, close to those of random classifiers.

Moreover, all performances of classifiers including a specific index were considered to get a deeper insight into which information allowed to get lower errors in the average. Considering MLP classifiers with 3 or 4 or 5 inputs, DET was always the index obtaining in the average less misclassifications, followed by RR and STD_*y*_. Considering SVM classifiers, DET was the index most included in optimal 3 inputs classifiers, whereas MNF allowed to get maximum average performances with 4 and 5 inputs (followed by RR, DET, and STD_*y*_). 

Finally, the best classifiers in dark conditions (the 6 classifiers in Table 4) were used to classify a set of test data, instead of a single value. Each classifier was trained and validated on a portion of data (randomly chosen for 20 times) and tested on the remaining data, which was a set of 2–6 samples. The mean error was quite high, in the range of 20%–30% for MLP and 30%–50% for SVM. The error was increasing as the number of samples to be classified increased (and, as a consequence, as the number of training data decreased). 

## 4. Discussion

This work aimed to investigate the ANS, through the characterization of the dynamics of the oscillations of the pupil of healthy adults in stationary conditions. A simple and noninvasive method is proposed. Sophisticated systems are available for the investigation of pupil dynamics [35]: high resolution cameras (about 1 megapixels) with around 0.5–1 kHz of sampling frequency even allow for the accurate investigation of microsaccadic movements [36]. The pupillogram is here investigated using a low cost recording system (less than 5.000 euro versus up to 40.000 euro needed for sophisticated devices [37]). Moreover, short experiments were conducted (1 minute long, compatible for a clinical investigation). The low sampling frequency and resolution (in terms of number of pixels) precluded the possibility of investigating fine details of pupil dynamics, like microsaccades. Nevertheless, the size and position of the pupil could be estimated and the indexes extracted from such time series allowed to identify with some confidence the conditions of stimulation of the ANS, even when the stimulus was very low. The HDO is proposed as a weak stimulus of the sympathetic system. In this work, we were able to distinguish it from the RP condition considering data recorded in darkness and using appropriate (linear and nonlinear) descriptors.

In stationary stimulation conditions, we assume that the physiological system is autonomous (the vector field determining the dynamics of the state variables of the system is constant in time; see Section 2.5). We can expect that, in such conditions, a sort of stationarity characterizes the time series. Nevertheless, we expect that the statistical properties of the pupillogram are neither constant in time nor simply periodic. Indeed, the ANS controls other physiological systems which show nonlinear behaviour; for example, the heart rate, which may display complex nonlinear dynamics, including deterministic chaos [38]. Thus, even pupil dynamics could be determined by nonlinear deterministic rules determining a nonlinear and chaotic behaviour. The analysis of recurrences of the dynamics is important to assess a nonlinear and possibly chaotic behaviour, so that we considered RQA.

 Obviously, a primary stimulus affecting pupil dynamics is light, which determines a large variation of most of the indexes that we recorded from pupillogram. For example, reduction of pupil size and of the range of movements were observed comparing data recorded in light with those acquired in darkness conditions. Moreover, each event (physical or mental), which is able to determine muscle activation or mental arousal, elicits a response of the sympathetic component of the ANS which involves an increase in pupil size [39]. Our results confirm this indication under darkness condition, showing that the HDO is to be considered as a physiological activation of the sympathetic component of the ANS.

Many biological signals are characterized in stationary conditions by fluctuations in time of their absolute values which often have nonlinear characteristics. These fluctuations have been studied by parameters derived from RQA (as RR and DET). Some studies showed that, in healthy subjects, more demanding physiological performance implies a reduction of determinism of signals reflecting the control of ANS (see e.g., [40]). On the contrary, in pathological conditions or aging, the dynamics of biological signals showed lower complexity and an increase in determinism [27]. In agreement with the aforementioned works, our data indicate that, under physiological conditions, autonomic and neuromuscular systems that control the dynamics of the pupil and ocular mobility respond simultaneously, although they are not necessarily correlated, in response to occlusion and light. Nonlinear parameters show a reduction of RR and DET, while, at the same time, there is an increase of the MNF of pupil oscillations and movements. Moreover, the dynamics of pupil oscillations in darkness show a reduction of the percentage energy of low frequency components (*F*_Low_), probably related to a change of the rate of breath, that raises in conditions of increased stress [19].

Taken together, darkness data suggest that occlusal contact involves physiological activation of the sympathetic branch of the ANS (pupil dilation, reduced values of DET and RR, reduction of low frequency components) and the oculomotor system (reduction of DET_*xy*_, increased frequency of the movements, and simultaneous reduction of the excursion). This might suggest, as a speculative, a role in arousal of dental occlusion in preparation for general responses of the body.

Statistical analysis of linear and nonlinear indexes indicated significant average variations consistent with our expectations and with literature. As a further step, individual investigation was here performed testing the possibility of characterizing the single signal, without considering paired recordings. We were specifically interested in discriminating between RP and HDO, which determines a slight stimulus to ANS. More indexes were needed to get acceptable performances in the discrimination of the two conditions. Here, it is important to understand if nonlinear indexes provide additional information on pupil dynamics with respect to linear ones. We tested the importance of the indexes by studying the performances in the classification of RP and HDO on the basis of them. We found that the discrimination of the two conditions is simpler by considering data recorded in darkness. Probably, light is a too strong stimulus to the ANS with respect to HDO. Optimal performance of classification in darkness condition was obtained including nonlinear information in the set of features, which also enclose the mean size and the vertical displacement, as linear descriptors.

Finally, our data suggest that the dynamics of spontaneous pupil size oscillation and of eye movement indicate the physiological response of ANS to “stress” conditions, such as HDO and stationary lighting. In the first case especially, if our data will be confirmed, the investigation of RQA indexes from pupillometry could be a simple, fast, and noninvasive method to study disorders related to dysregulation of the ANS. For example, patients suffering from Temporo Mandibular Dysfunction (TMD) show impairment of ANS balance between sympathetic and parasympathetic path and dental occlusal contact could trigger chronic pain [41, 42]. Preliminary results obtained by our group using linear analysis of pupillometry indicate that the adrenergic function is dysregulated in patients with TMD [29]. This paper indicates that nonlinear information is additional to that provided by linear indexes and allows to improve the characterization of pupil dynamics. Work is in progress to assess the variation of RQA indexes in healthy subjects and in patients under different conditions. For example, a preliminary study indicated the possibility of discriminating patients (TMD or patients with obstructive sleep apnea syndrome, OSAS) from a control group investigating linear and RQA pupil indexes [43].

Here, we focused only on nonlinear indexes extracted from RQA. Such indexes are related to other nonlinear information: RR is related to many nonlinear indexes, which are usually based on the study of recurrences (e.g., correlation dimension and fractal dimension [31]); DET indicates how much nearby trajectories stay close to each other and is related to Lyapunov exponents. However, the use of other nonlinear indexes (e.g., fractal dimension or entropy) could provide additional information on pupil dynamics and is suggested for future studies. 

## 5. Conclusions

The pupillogram reflects the state of the autonomous nervous system (ANS). A simple, short, low cost experiment (adequate for a clinical setting) is proposed, based on the investigation of pupil dynamics in darkness with the jaw in rest position (RP) or during a habitual dental occlusion (HDO). The joint analysis of linear and RQA indexes extracted from the pupillogram is sensitive enough to discriminate between these two conditions, determining weakly different stimulations of the ANS.

## Figures and Tables

**Table 1:** Mean ± standard deviation of indexes.

Index	RP	RP	HDO	HDO
	Darkness	Light	Darkness	Light
Size (pixel)	7020 ± 1484	3751 ± 1808	7139 ± 2373	4047 ± 1608
MNF (Hz)	0.337 ± 0.246	0.239 ± 0.059	0.314 ± 0.197	0.231 ± 0.091
*F*_Low_ (%)	27.2 ± 12.1	19.6 ± 8.2	24.4 ± 11.9	20.8 ± 15.7
*F*_High_ (%)	16.9 ± 7.9	19.2 ± 10.5	17.8 ± 8.6	18.5 ± 8.4
MNF_*x*_ (Hz)	0.165 ± 0.061	0.298 ± 0.142	0.204 ± 0.123	0.264 ± 0.172
MNF_*y*_ (Hz)	0.225 ± 0.101	0.452 ± 0.270	0.249 ± 0.167	0.490 ± 0.256
STD_*x*_ (pixel)	8.518 ± 3.914	3.675 ± 4.695	6.657 ± 3.592	3.255 ± 1.740
STD_*y*_ (pixel)	5.134 ± 3.602	3.732 ± 4.069	4.850 ± 2.729	2.843 ± 2.525
RR (%)	25.2 ± 19.9	8.9 ± 6.8	12.4 ± 10.2	7.9 ± 3.6
DET (%)	87.5 ± 19.7	87.6 ± 7.5	77.9 ± 19.9	89.6 ± 5.6
RR_*xy*_ (%)	2.7 ± 6.0	4.3 ± 11.8	2.8 ± 6.0	1.2 ± 0.9
DET_*xy*_ (%)	16.7 ± 24.0	22.5 ± 22.6	15.8 ± 24.0	14.7 ± 7.8

**Table 2:** Two-sided Wilcoxon signed rank test (*P* values).

Index	RP light versus darkness	RP versus HDO (light)	RP versus HDO (dark)	HDO darkness versus light
Size (pixel)	***0.00001***	0.36	***0.051***	***0.00001***
MNF (Hz)	***0.032***	0.24	0.41	***0.05***
*F*_Low_ (%)	***0.057***	0.15	***0.048***	***0.003***
*F*_High_ (%)	0.21	0.55	0.23	***0.032***
MNF_*x*_ (Hz)	***0.020***	0.75	0.06	0.93
MNF_*y*_ (Hz)	***0.007***	0.061	0.84	***0.00007***
STD_*x*_ (pixel)	***0.001***	0.17	0.11	***0.001***
STD_*y*_ (pixel)	0.071	0.12	***0.037***	***0.0001***
RR (%)	***0.0002***	0.15	***0.010***	***0.006***
DET (%)	0.85	0.22	***0.007***	***0.003***
RR_*xy*_ (%)	0.79	0.11	0.25	0.73
DET_*xy*_ (%)	0.17	***0.019***	0.54	0.09

bold italic numbers correspond to significant differences.

**Table 3:** Classification of RP and HDO in light conditions.

RP versus HDO in light conditions	MLP		SVM	
	Input indexes	Error	Input indexes	Error
3 inputs	MNF *F*_Low_ MNF_*y*_	46.2%	RR MNF MNF_*y*_	26.9%
4 inputs	Size MNF *F*_Low_ STD_*x*_ RR DET *F*_Low_ STD_*y*_	46.2%	RR MNF *F*_Low_ STD_*x*_	34.6%
5 inputs	RR DET MNF *F*_Low_ STD_*x*_	50%	DET MNF *F*_Low_ MNF_*y*_ STD_*y*_	34.6%

Set of input features (with 3, 4, or 5 inputs) providing best classification of RP and HDO in light.

**Table 4:** Classification of RP and HDO in darkness conditions.

RP versus HDO in light conditions	MLP		SVM	
	Input indexes	Error	Input indexes	Error
3 inputs	Size DET STD_*y*_	19.2%	RR MNF MNF_*y*_	26.9%
4 inputs	Size RR DET STD_*y*_	19.2%	Size DET MNF STD_y_	26.9%
5 inputs	Size RR DET *F*_Low_ STD_*y*_	15.4%	Size DET MNF MNF_*y*_ STD_*y*_	26.9%

Set of input features (with 3, 4, or 5 inputs) providing best classification of RP and HDO in darkness.
